# Extrapulmonary Small Cell Carcinoma: A Single-Institution Review of Brain Metastases, Treatment Paradigms, and Patient Outcomes

**DOI:** 10.7759/cureus.76974

**Published:** 2025-01-05

**Authors:** Sarah E Glynn, Rachel Shenker, Niema B Razavian, Zachary Patel, Cole Steber, Claire M Lanier, Joshua Farris, Michael Farris, Michael Chan, Ryan T Hughes

**Affiliations:** 1 Radiation Oncology, Wake Forest University School of Medicine, Winston-Salem, USA; 2 Radiation Oncology, Duke University Hospital, Durham, USA

**Keywords:** brain metastases, carcinoma, extrapulmonary, gamma knife, neuroendocrine, prophylactic cranial irradiation, radiotherapy, small cell, stereotactic radiosurgery, whole-brain radiotherapy

## Abstract

Purpose

Small cell carcinoma of the lung (SCLC) often presents with brain metastases, with most patients developing them within a few years of diagnosis. Prophylactic cranial irradiation (PCI) is commonly recommended. Extrapulmonary small cell carcinoma (EPSCC) is rare, and its metastatic pattern is not well understood. This study reviews brain metastases in EPSCC patients at a single institution, focusing on management and overall survival (OS).

Materials

We identified EPSCC patients and analyzed their characteristics, treatment, and outcomes. Brain metastases were assessed through diagnostic imaging. Extracranial progression-free survival (ePFS) was defined as the time from diagnosis to progression outside the brain, while OS was defined as the time from diagnosis to death from any cause. Kaplan-Meier methods and log-rank tests were used for time-to-event analyses, and the cumulative incidence of brain metastasis was estimated with the competing risk of death. Statistical significance was set at p < 0.05.

Results

Of the 68 EPSCC patients with a median follow-up of 7.1 months, 66% were male with a median age of 68 years old. Common primary sites included genitourinary (32%) and gastrointestinal/hepatobiliary (22%). Brain metastases occurred in 12 patients (18%): five at diagnosis and seven during follow-up. The treatment of brain metastases varied, with four patients receiving whole-brain radiotherapy (WBRT), two receiving stereotactic radiosurgery (SRS), and one receiving both WBRT and SRS. The median OS was 10.0 months, with no significant survival difference between patients with (10.8 months) and without (9.4 months) brain metastases (p = 0.89).

Conclusion

EPSCC has a lower incidence of brain metastases than SCLC, and brain metastases do not significantly impact OS. Further research on brain imaging, PCI, and management strategies is warranted.

## Introduction

Extrapulmonary small cell carcinoma (EPSCC) is a rare malignancy, accounting for 5% of all small cell carcinoma diagnoses, with 1,000 cases reported annually in the United States [1]. Unlike small cell lung cancer (SCLC), EPSCC has been reported in various sites outside the lung, including the genitourinary and gastrointestinal tracts, head and neck, gynecologic organs, and skin [2,3]. Despite originating in different sites, both EPSCC and SCLC are poorly differentiated small round blue cell tumors with neuroendocrine features, clinically aggressive, and associated with early distant metastatic spread [4]. There is no universally accepted standard treatment for EPSCC, and management typically follows extrapolated guidelines based on the site of involvement [5].

Because 50%-80% of SCLC patients develop brain metastasis, prophylactic cranial irradiation (PCI) has historically been used to mitigate the morbidity and mortality associated with CNS progression [6]. However, recent studies suggest PCI may not be necessary for all SCLC patients [7]. Surveillance with magnetic resonance imaging (MRI) and delaying treatment until brain metastases occur do not appear to impact overall survival (OS) [7]. Additionally, more focal irradiation techniques, such as stereotactic radiosurgery (SRS), have proven effective for patients with brain metastasis from SCLC [8]. Whether these practices apply to EPSCC patients remains unclear. Due to the rarity and heterogeneity of EPSCC, it is uncertain whether this malignancy shares the same propensity for CNS spread as SCLC.

This study presents a single-institution retrospective review of the clinical outcomes in EPSCC patients. Given the uncertainty surrounding surveillance versus PCI in EPSCC, we focused our analysis on the incidence of brain metastases, their management, and their impact on OS.

## Materials and methods

Data acquisition

This study was conducted at Atrium Health Wake Forest Baptist, Winston-Salem, and was approved by the Wake Forest Health Sciences Institutional Review Board (IRB00049750). Patients with EPSCC diagnosed between 2010 and 2020 (n = 82) were identified through a query of an institutional database. Patients without available records or follow-up after initial diagnosis were excluded (n = 14), resulting in a final cohort of 68 patients. Patient characteristics, treatment paradigms (including chemotherapy, surgery, and/or radiotherapy), and clinical outcomes were determined by reviewing electronic medical records. The presence of brain metastasis was determined by reviewing all available MRI and computed tomography (CT) examinations of the brain, both at diagnosis and during follow-up. Pathologic confirmation was not required if prior evidence of distant metastatic disease was present.

Patient management and response assessment for patients with brain metastases

Treatment of brain metastases included either whole-brain radiotherapy (WBRT) or SRS. WBRT was administered using opposed lateral fields, delivering a dose of 30 Gy in 10 fractions. SRS was performed in a single fraction, with doses ranging from 18 to 22 Gy prescribed to the 80% isodose line (IDL), in accordance with our institutional standard [9,10]. The posttreatment baseline MRI was performed 6-8 weeks after completion of radiotherapy, and then every three months thereafter for at least two years.

Statistics

Descriptive statistics were calculated using counts (frequencies) and medians (ranges) where appropriate. The time from initial EPSCC diagnosis to each outcome of interest was estimated using the Kaplan-Meier method, with patients censored at the date of death or last follow-up. Extracranial progression-free survival (ePFS) was defined as the time from diagnosis to disease progression outside the brain, while OS was defined as the time from diagnosis to death from any cause. Differences in time-to-event outcomes between groups were assessed using the log-rank test. The cumulative incidence of brain metastasis was estimated from the date of diagnosis, with death without brain metastasis considered a competing risk [11]. Statistical analyses were performed using R version 3.6 (R Foundation for Statistical Computing, Vienna, Austria). p-values less than 0.05 were considered statistically significant.

## Results

Patient characteristics

From 2010 to 2020, 68 patients with EPSCC were diagnosed at our institution. Patient characteristics are summarized in Table 1. The majority of patients were male (66%), with a median age at diagnosis of 68 years, an ECOG status of 1 (49%), and were nonsmokers (73%). The primary sites of EPSCC included genitourinary (32%), gastrointestinal/hepatobiliary (22%), head and neck (13%), and unknown (21%). The most frequently involved organs were the bladder (15%), prostate (15%), and pancreas (6%). At diagnosis, 41 patients (60%) had metastatic disease, defined as disease outside the primary site or regional lymph nodes.

**Table 1:** Patient characteristics ECOG: Eastern Cooperative Oncology Group, MRI: magnetic resonance imaging, CT: computed tomography.

		n (%)
Age (median, (range))		68 (59-75)
Sex		
	Male	45 (66.2%)
	Female	23 (33.8%)
Race		
	White	56 (82.4%)
	Asian	1 (1.5%)
	African American	10 (14.7%)
	Unknown	1 (1.5%)
Tobacco use		
	Never smoker	49 (73.1%)
	Current smoker	18 (26.9%)
ECOG performance status		
	0	34 (66.7%)
	1	17 (33.3%)
Metastatic at diagnosis		
	Yes	41 (60.3%)
	No	27 (39.7%)
Primary tumor site		
	Genitourinary	22 (32.4%)
	Gastrointestinal	15 (22.1%)
	Unknown	14 (20.6%)
	Head and Neck	9 (13.2%)
	Gynecologic	6 (8.8%)
	Breast	2 (2.9%)
Brain imaging prior to treatment		40 (58.8%)
	MRI brain	35 (87.5%)
	CT head	5 (12.5%)

Treatment of extracranial disease

Definitive treatment was administered to 55 patients (81%). Treatment details are summarized in Table 2. Modalities included chemotherapy (n = 48), radiotherapy to the primary site (n = 23), and surgery to the primary site (n = 18), with 43 receiving more than one treatment type. Chemotherapy regimens included carboplatin/etoposide (50%), cisplatin/etoposide (31%), and other regimens (19%).

**Table 2:** Treatment details WBRT: whole-brain radiotherapy, SRS: stereotactic radiosurgery, CNS: central nervous system.

		n (%)
Treatment received		55 (80.9%)
Chemotherapy		48 (70.6%)
	Cisplatin/etoposide	15 (31.2%)
	Carboplatin/etoposide	24 (50.0%)
	Other	9 (18.8%)
Radiation to primary site		23 (34.3%)
Surgery to primary site		18 (28.1%)
Multimodality		43 (63.2%)
Brain metastases		
De novo		5 (7.4%)
	WBRT	4 (5.9%)
	SRS	0 (0%)
Subsequent		7 (11.1%)
	SRS	2 (2.9%)
	SRS and WBRT	1 (1.5%)
Follow-up CNS imaging		37 (54.4%)

OS and extracranial progression

**Figure 1:**
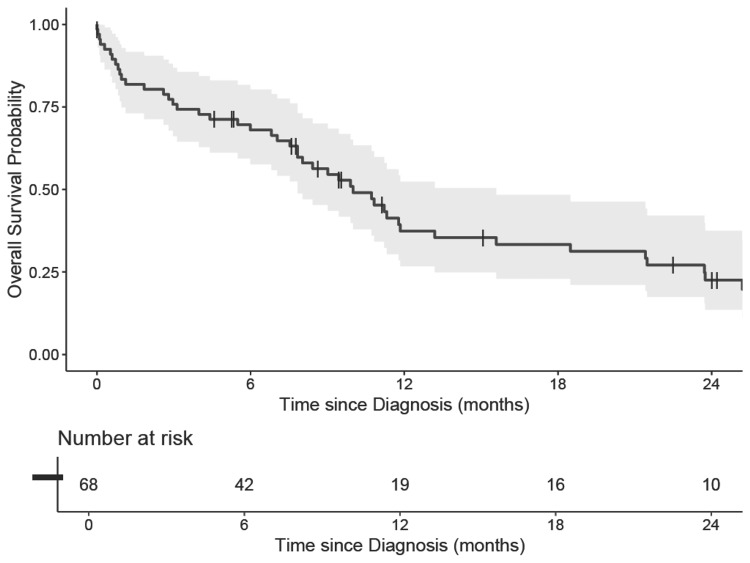
Overall survival for patients diagnosed with extrapulmonary small cell carcinoma

**Figure 2:**
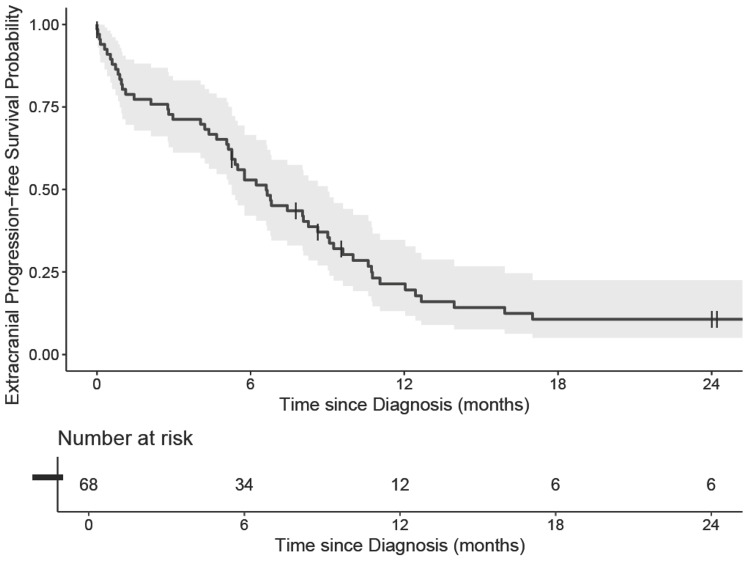
Extracranial progression-free survival in patients diagnosed with extrapulmonary small cell carcinoma

The median follow-up after diagnosis was 7.1 months (95% CI, 0.0-44.6). OS for the entire cohort is shown in Figure 1. Of 68 patients, 49 died. The median OS for the entire cohort was 10.0 months (95% CI, 7.8-15.6). Kaplan-Meier estimates of OS at one and two years were 37% (95% CI, 27%-52%) and 23% (95% CI, 14%-38%), respectively. There was no association between the primary site of EPSCC and OS (p = 0.15) or ePFS (p = 0.64). Fifty patients experienced disease progression; ePFS was 21.4% (95% CI, 13.2-34.7) at one year and 10.7% (95% CI, 5.1-22.5) at two years, as shown in Figure 2.

Development and treatment of brain metastases

Diagnostic imaging was available for 59% of patients: 40 patients (59%) had diagnostic brain imaging at the time of diagnosis and 37 (54%) during follow-up. Among those with diagnostic brain imaging, five (13%) had brain metastases. Of these patients, four received WBRT, and one received no treatment. Among the 37 patients with follow-up brain imaging, seven (19%) developed brain metastases during the follow-up period. Treatment for brain metastases detected during follow-up included SRS (n = 2), WBRT and SRS (n = 1), and no brain-directed therapy (n = 4). The five patients who did not receive brain metastases treatment were referred to hospice care. In total, 12 of 68 (18%) patients developed brain metastases at diagnosis or during follow-up, while no brain metastases were observed in the remaining 56 patients (82%).

**Figure 3:**
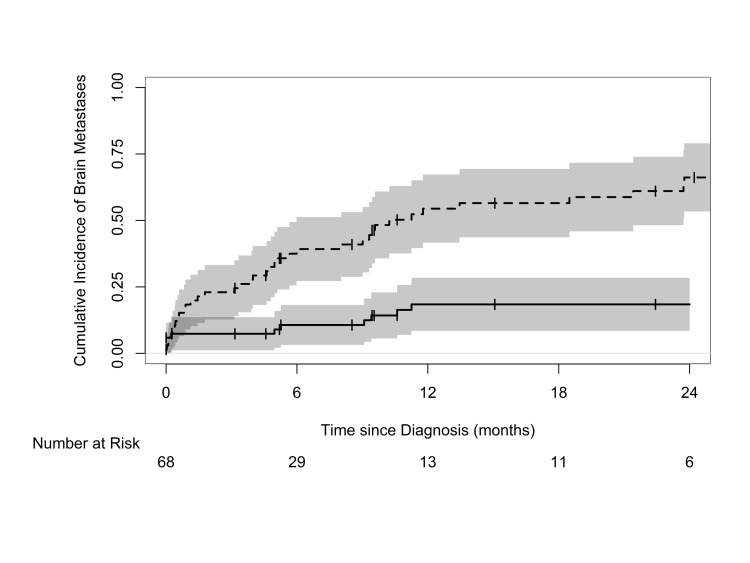
Cumulative incidence of brain metastases (solid line) and death without brain metastases (dashed line) in patients diagnosed with extrapulmonary small cell carcinoma

The cumulative incidence of brain metastasis, with death as a competing risk, is depicted in Figure 3. The one- and two-year cumulative incidence of brain metastases were both 18%. The cumulative incidence of death without brain metastasis was 55% at one year and 66% at two years. There was no significant difference in median OS between patients who developed brain metastasis at any point (10.8 months) and those who did not (9.4 months; log-rank test, p = 0.89).

## Discussion

In this retrospective study of patients with EPSCC, we identified comparably low rates of brain metastases, as well as high rates of progression or death within two years. This pattern of failure is fundamentally different from what would be expected in SCLC and has important implications for the utility of PCI.

Several prior reports have contributed to describing the natural history of EPSCC. The disease is known to have a poor prognosis, particularly in patients with metastatic disease at the time of diagnosis [12]. Five-year OS has been reported to range from less than 5% to 40%, consistent with our findings [13]. Similar to SCLC, EPSCC generally responds to chemotherapy, as would be expected with high-grade neuroendocrine carcinoma, but these responses are typically transient [5]. Given the rarity of EPSCC, it remains unclear whether its natural history mirrors that of SCLC. In the limited published series of EPSCC, differences in prognosis appear to exist depending on the site of origin, with patients with gynecologic primaries having the best outcomes [12].

Due to a paucity of data, standard treatment guidelines for EPSCC have not been clearly delineated. However, population-based studies suggest that surgical resection may benefit patients with localized disease, and radiotherapy may be beneficial for those with localized gastrointestinal primaries [14]. In the present series, 34% of patients received radiotherapy at the primary site, and 28% underwent surgical resection of the primary tumor. Seventy percent of the patients received predominantly platinum-based chemotherapy. The treatment paradigms utilized in our study align with common practices in the United States. In general, patients with metastatic disease at presentation are typically treated with platinum-based combination chemotherapy [15]. Those with localized disease are often treated with chemotherapy in combination with some form of tumor-directed therapy, likely extrapolated from treatment paradigms for more common malignancies at each primary site, though the benefit of further adjuvant therapy remains unclear.

An important clinical question that remains unanswered concerns the incidence of brain metastases in EPSCC, given the predilection of SCLC to metastasize early and frequently to the brain [16]. Multiple prior series have demonstrated that both histology and the primary cancer site can influence the likelihood of developing brain metastases [17,18]. In lung cancers, early studies demonstrated that PCI could potentially improve survival in patients with SCLC, where the chest disease responded to treatment [6,19]. While this improvement in survival was hypothesized to result from treating occult brain disease [7], the question has recently been asked whether proper brain staging prior to PCI might allow for more focal treatment of brain metastases. If EPSCC were shown to have a similar propensity for CNS spread as SCLC, MRI surveillance, PCI, and reserving stereotactic radiosurgery (SRS) for salvage therapy could be relevant aspects of EPSCC management. Prior series suggest that PCI may not be necessary for EPSCC but do not rigorously document the rate of CNS seeding by EPSCC [20].

In the present series, the overall incidence of brain metastases in EPSCC appears somewhat lower than what would be expected for SCLC. It is estimated that approximately 20% of patients with SCLC will have brain metastases at diagnosis and more than 50% will ultimately develop brain metastases [21]. In the present series, only 18% of patients developed brain metastases, and 87% died by the last follow-up. In a recent series by Ferro et al., only two of the 41 patients (5%) with EPSCC developed brain metastases [20], which is lower than the 18% observed in our study. This difference could be influenced by several factors, including the frequency of brain imaging during workup and surveillance, treatment variations, and competing mortality rates without identification of CNS disease.

SCLC has not only demonstrated early spread to the brain but also a propensity for multifocal disease and high rates of continuous brain seeding [22]. This has led to the common use of WBRT as a standard first-line treatment for brain metastases from SCLC [23]. As a result, SRS has traditionally been reserved for salvage therapy [24]. Recently, a multi-institutional analysis demonstrated favorable local and distant brain control after upfront SRS that is comparable to what is seen in non-SCLC diseases [8,25]. WBRT improved time to CNS progression, but it did not affect survival, and it may be most beneficial in patients with more than five brain metastases. For EPSCC, the optimal upfront treatment for brain metastases remains unknown. While the number of patients in the present series is small, the patients were generally managed using a standard brain metastasis paradigm from which SCLC has commonly been excluded: patients with oligometastatic brain disease were often treated with SRS, while WBRT was reserved for more numerous lesions. Due to the small size of the present series, only three patients underwent upfront SRS for brain metastases. However, these patients did not experience rapid distant brain failure or require rapid WBRT rescue.

There are several limitations of the present series. Given the retrospective nature of the study, it is subject to selection biases and confounding effects of practice patterns at a tertiary academic center. The number of patients in the series is small, limiting the power of the series to detect differences in subpopulations. Due to these limitations, the data presented in the present study are primarily hypothesis-generating and will need to be validated by independent datasets. However, a multi-institutional or cooperative group prospective study may not be feasible for this rare disease. Additional focus on multi-institutional, collaborative research efforts in this disease is needed to better understand the nature of EPSCC and to establish more standardized management.

## Conclusions

In contrast to SCLC, the development of brain metastases in patients diagnosed with EPSCC is uncommon. Brain metastases, when observed, did not impact OS for patients with EPSCC. Further studies investigating the role of brain imaging, PCI, and the optimal upfront management of brain metastases are warranted.
